# The Use of *Acartia tonsa* Nauplii during the First Days of Feeding on the Ontogeny of the Digestive System of Greater Amberjack (*Seriola dumerili* Risso, 1810)

**DOI:** 10.1155/2024/1826300

**Published:** 2024-04-01

**Authors:** Katerina Loufi, Ioannis E. Papadakis, Pavlos Makridis

**Affiliations:** ^1^Department of Biology, University of Patras, Rio Achaia, Patras, Greece; ^2^Hellenic Centre for Marine Research, Institute of Marine Biology, Biotechnology and Aquaculture, Iraklion, Greece

## Abstract

The effect of feeding greater amberjack with copepod nauplii (*Acartia tonsa*) on the ontogeny of the digestive system was observed until 40 days after hatching (DAH). Copepods are part of the diet of fish larvae in nature, and they are rich in highly unsaturated fatty acids and free amino acids that enhance the digestive capacity of the fish. In a marine hatchery, four cylindroconical tanks of 2,700 L were stocked with about 150 × 10^3^ greater amberjack larvae (*Seriola dumerili*) in each. The larvae were initially fed from 3 to 17 DAH in two tanks with copepod nauplii and rotifers (*Brachionus* sp.; Copepods group), while in the other two tanks, they were fed only with rotifers (Control group) during the same period. All the tanks were fed with rotifers (3–27 DAH), *Artemia* nauplii (12–22 DAH), enriched *Artemia* metanauplii (20–30 DAH), and formulated diet (25–40 DAH). Fish samples were taken regularly (every 2 or 4 days) for histological analysis and every day for the measurement of total length (TL). The TL was 3.7, 4.5 ± 0.1, 6.1, 11, 17.3 ± 0.1 and 20.3 ± 2.3 mm at 4, 10, 16, 22, 30, and 40 DAH, respectively. Copepod-fed fish showed higher TL in the last 2 days of the trial (*p*  < 0.05), while mortality rates were lower in the beginning of the trial 10–17 DAH, (*p*  < 0.05). In addition, copepods-fed fish had less skeletal deformities (*p*  < 0.05). Pyloric caeca appeared earlier in the Copepods group compared with the Control, while the length and surface of the villi, the abundance of goblet cells/100 *μ*m of intestine length, and the area covered with lipid vacuoles in the liver were significantly higher in the Copepods group (*p*  < 0.05). We can conclude that the use of copepods in the diet of the greater amberjack larvae can improve the ontogeny of the digestive system.

## 1. Introduction

Greater amberjack (*Seriola dumerili;* Risso 1810) is a marine pelagic species [1], which has a rapid growth rate [2], very good flesh quality [3], and therefore has great potential in the industry of aquaculture [4]. There is only one extensive study comparing the ontogeny (organogenesis and development) of the digestive system of greater amberjack, in which the species was cultured in two different rearing conditions (semi-intensive and intensive system with water recirculation) [5]. More studies about the ontogeny of the digestive system have been carried out on other *Seriola* species, such as *S. lalandi* [6, 7] and *S. rivoliana* [8].

The digestive system is responsible for breaking down and absorbing nutrients [9]. During the first developmental stages and until the end of metamorphosis, the digestive system of the fish undergoes several changes regarding its morphology and functionality [10–12]. The development of the digestive system influences the proper development of the skeleton and the nervous system and is also important during the weaning period where there is a gradual transition of fish from live to formulated feed. To accomplish successful weaning with minimum mortalities, a fully functional stomach is required. If the weaning period does not occur at the correct time, and the digestive system is not organized properly, the mortality rates increase since the larvae cannot digest the formulated diet [13].

The knowledge of the ontogeny of the digestive system is a useful tool for a better understanding of the nutritional requirements of the species in different developmental stages. It is also useful for optimizing feeding protocols for each species. Greater amberjack is a fast-growing species, and thus the use of feeding protocols developed for other species, such as sea bream and sea bass, cannot be applied in its rearing.

The efficiency of the digestive process and the activity of the digestive enzymes depend on many factors, such as water temperature and diet [14–16]. Copepods are important part of the natural diet of greater amberjack during the first developmental stages and up to the juvenile stage [17]. It has been found that the nutritional profile of the copepods is more adequate than that of rotifers and *Artemia*, with a higher abundance of highly unsaturated fatty acids (HUFAs), such as docosahexaenoic acid (DHA) and eicosapentaenoic acid (EPA) [18]. These fatty acids are accumulated in phospholipids, and thus there is no need for enrichment. The absence of enrichment products can decrease the lipid layer on the surface of rearing tanks, therefore improving the conditions for the inflation of the swim bladder [18]. Phospholipids requirements are related to the larval age and the degree of digestive system development [19, 20]. A recent study showed that the higher the levels of DHA in the diet of fish larvae, the higher the activity of digestive enzymes (protease, lipase, alkaline phosphatase, and leucine), resulting in a great improvement in the digestive capacity of the fish larvae [21]. Fatty acid content could be involved in increasing the area covered with lipid vacuoles in both the posterior intestine and liver [22], which are fat depositions that respond and are rapidly depleted during periods of starvation or diet not adapted to the requirements of the fish [23, 24]. An additional advantage of the copepods is that they are rich in free amino acids, which can enhance the feeding behavior of the fish larvae [18]. Amino acids are important for anabolic and catabolic processes which are associated with the growth and development of fish [25–27]. Dietary amino acids can be absorbed easily and more efficiently by the fish larvae when coming from natural and not enriched zooplankton and without prior digestion [28–30]. Finally, copepods contain important micronutrients, such as vitamin C, vitamin E, calcium, copper, iron, magnesium, selenium, and zinc which are beneficial for the proliferation of goblet cells. The concentration of these micronutrients is higher in copepods compared with *Artemia* nauplii and enriched rotifers and *Artemia* metanauplii [31]. Goblet cells are mucus-secreting cells. The amount of mucus affects lubrication, osmoregulation, and the efficiency of the immune system [32].

The aim of this work was to study organogenesis and development of the digestive system in greater amberjack larvae after using *Acartia tonsa* nauplii during the first days of exogenous feeding.

## 2. Materials and Methods

### 2.1. Larval Rearing and Live Food

Greater amberjack larvae and juveniles were reared in a commercial marine fish hatchery, Galaxidi Marine Farm S.A., Greece. Amberjack yolk-sac larvae were stocked in four cylindroconical tanks of 2,700 L at a density of about 55 larvae/L. Seawater temperature was initially 23°C and was gradually increased to 26°C, pH was 8, salinity 36 g/L, and the dissolved oxygen saturation level was 85%–95%. The larvae were kept in dark conditions until 3 days after hatching (DAH). Light intensity was initially 800 lx and was increased to 1500 lx at 15 DAH, reduced to 1,000 lx at 20 DAH, and remained stable thereafter until the end of rearing at 40 DAH. The water renewal was initiated at 3 DAH and gradually increased thereafter. Nitrate, nitrite, and mortality rates were monitored daily. Surface skimmers for the removal of the lipid film were placed at 7 DAH. Two feeding protocols were applied. In two tanks, fish larvae were fed with enriched rotifers (*Brachionus* sp.) from 3 to 27 DAH, newly-hatched *Artemia* sp. nauplii from 10 to 22 DAH, enriched *Artemia* sp. metanauplii from 20 to 30 DAH, and formulated feed from 25 DAH until the end of the experiment (40 DAH; Control group). Following the second feeding protocol, newly-hatched *A. tonsa* nauplii were added to the tanks from 3 to 17 DAH (Copepods group). After 17 DAH the feeding protocol was the same for both Control and Copepods group. The vitamin, mineral, and fatty acid content of the different live food organisms and the formulated diet used in the rearing trial are shown in Table 1. *A. tonsa* cysts were provided by CFEED AS, Norway. The cysts were rinsed with seawater and placed in cylindroconical tanks with intense aeration at 25°C and hatched after about 24 hr. Rotifers were enriched with DHA SELCO (INVE Aquaculture, S/A, Belgium) at 25°C. *Artemia* metanauplii were enriched with Easy DHA SELCO (INVE Aquaculture, S/A, Belgium) overnight. The formulated diet that was used, was the Caviar, Nature, BernAqua, Olen, Belgium. Live food was administered every 4 hr manually (six feedings per day) and the different types of food were administered to the tanks simultaneously. The formulated diet was administrated manually several times during the day and its amount was progressively increased. The feeding protocol of this trial is described in detail in Table 2.

### 2.2. Histological Analysis

Six larvae were sampled from each tank for histological analysis from 4 to 40 DAH, while for the measurement of the total length 15 fish were sampled from 1 to 40 DAH (Table 2). The fish were anesthetized with ethylene glycol monophenyl ether (0.2–0.5 mL/L, Merck, Germany) and fixed in 4% formaldehyde solution. Fish were dehydrated through a series of increasing concentrations of ethanol (70%–100%) and then they were embedded in a methacrylate resin (Technovit 7100®, Heraeus Kulzer, Germany). The mounted larvae were cut in 5-*μ*m-thick sections by use of a microtome (Leica SM2000R, Germany), stained with methylene blue (Sigma, Germany)/Azure II (Sigma, Germany)/basic Fuchsin (Polysciences, USA) [35], and examined with a light microscope (microscope Zeiss AX10 Image A2). The total length (TL) of the fish larvae, the length and surface of the villi and the abundance of goblet cells in the anterior intestine, as well as the percentage of the area covered with lipid vacuoles (ACLV) in the liver, were calculated by using an image analysis software (Image J, NIH, USA). A modified protocol from [36]) was used for the measurement of the length and surface of the villi, and the abundance of goblet cells in the intestinal epithelium. Six microphotographs were obtained from six different areas of the anterior intestine. The measurement of the length and surface of the villi was possible from day 4 of the experiment, while goblet cells appeared later. For the calculation of the abundance of goblet cells, a line by hand was drawn and then the length of this line was calculated in pixels. The pixels were converted into micrometers and then the number of goblet cells was calculated per 100 *μ*m of intestine length. To calculate ACLV (%) in the liver, six microphotographs from six different areas were obtained and converted to grayscale and then the area with lipid vacuoles in white was measured, while glycogen was manually excluded from the analysis according to Papadakis et al. [10].

### 2.3. Statistical Analysis

The Kolmogorov–Smirnov normality test was performed on the data of the length and surface of the villi, the abundance of goblet cells and the ACLV in the liver. As it showed that those data had not a normal distribution, they were transformed using square root and a one-way ANOVA was conducted, followed by the Games–Howell test, to check whether there were any differences in the ontogeny of the digestive system between the four tanks. Finally on the same data, an independent *t*-test at *p*  < 0.05 was performed to check if there were any statistically significant differences between the two feeding protocols. For the presentation of the results, raw data were used, and they are presented as mean ± SD. The data of the different histological indices (villi and surface of the villi, abundance of goblet cells, and ACLV in the liver) were normalized against total fish length to test the effect of copepods on the ontogeny of the digestive system of the greater amberjack larvae independently of the effect of growth. We divided thereby the results of every index in each fish sampled with its total length and apply the independent *t*-test at *p*  < 0.05. Thus, it was possible to determine whether the differences in the ontogeny of the digestive system of the two groups were due to the effect of a difference in growth or to the use of different feeding protocols.

## 3. Results

### 3.1. Zootechnical Observations

**Figure 1:**
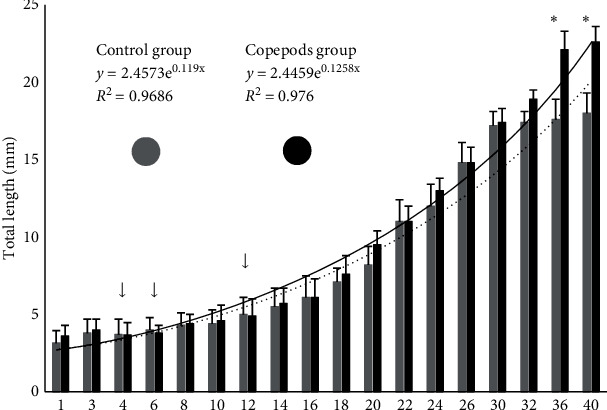
Total length (mm ± S.D.) of greater amberjack in both control (grey bars, *n* = 21 ind/tank) and copepods (black bars, *n* = 21 ind/tank) group. The dashed line shows the trend line for the Control group and the solid black line for the copepods group. Arrows (↓) denote the days when fish from the control group were larger than fish from the Copepods group and asterisks ( ^*∗*^) denote the days with statistically significant differences (*p*  < 0.05).

Fish larvae from the Copepods group showed a higher total length (mm) than the fish larvae from the Control group, on days 37 and 40 (*p*  < 0.05; Figure 1). Growth performance adjusted exponentially to the equations: *y* = 2.4573e^0.119x^, *R*^2^ = 0.9686 for the Control group and *y* = 2.4459e^0.1258x^, *R*^2^ = 0.976 for the Copepods group.

**Figure 2:**
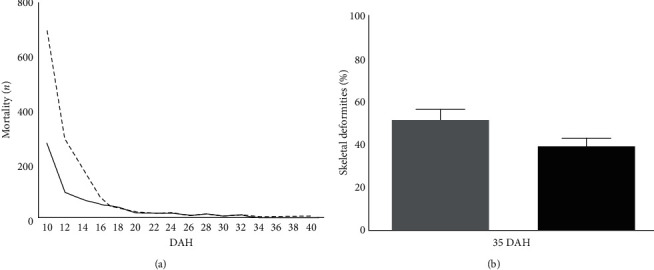
(a) Mortality (*n*) from 10 to 40 DAH (*p*  < 0.05). The dashed line shows the mortality in the Control group and the solid black line for the Copepods group. (b) Skeletal deformities on 35 DAH (*p*  < 0.05) of the fish larvae of greater amberjack between the Control (grey) and the Copepods (black) group.

In addition, the mortality rate of the fish from the Control group was higher than in the fish from the Copepods group from 10 to 17 DAH (*p*  < 0.05). During the rest of the experiment, the mortality rate in the two groups was similar (Figure 2(a)). Finally, skeletal analysis on 35 DAH showed significant differences in the appearance of deformities in the juveniles of the two different feeding protocols (Figure 2(b)).

There was no evidence of cannibalism or aggression among the fish in the rearing tanks.

### 3.2. Ontogeny of the Digestive System

**Figure 3:**
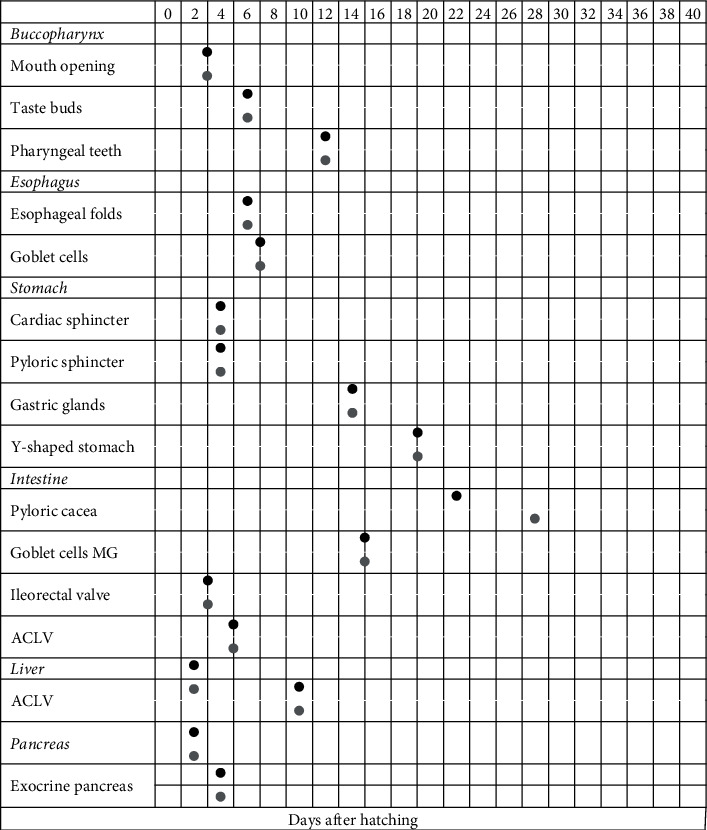
Schematic representation of the appearance of the main developmental structures examined in the greater amberjack larval digestive system (grey solid circles indicate the Control group, black solid circles indicate the Copepods group), as a function of days after hatching (DAH, horizontal axis).

At hatching, greater amberjack has an undifferentiated and undeveloped digestive tract positioned dorsally to the yolk sac, without a formed mouth and anus. The main structures of the digestive system of greater amberjack larvae appeared simultaneously in both the Control and the Copepods group. The only case where the time of appearance was different between the two groups was in the case of the pyloric caeca of the intestine. More specifically, the early hepatic cells appeared 2 DAH and were initially located behind the yolk sac and under the anterior intestine. The pancreatic cells also appeared at 2 DAH as an undifferentiated tissue and the differentiation of the endocrine and exocrine regions did not begin before 4 DAH (3.7 ± 0.01 mm TL). The mouth of the fish larvae opened at 3 DAH (3.9 ± 0.1 mm TL), at the same time as the formation of the ileorectal valve in the intestine. The cardiac and pyloric sphincters formed 4 days after hatching. During 6 and 7 DAH (3.9 ± 0.1 mm TL), the taste buds and the esophageal folds appeared as well as the first goblet cells in the esophagus. The week between the 12^th^ and 19^th^ day after hatching was particularly important for the ontogeny of the digestive system since pharyngeal teeth (12 DAH, 5 mm TL), gastric glands (14 DAH, 5.6 ± 0.1 mm TL), and goblet cells in the intestine (15 DAH) appeared. Later the stomach acquired the characteristic Y-shape which is an indication that we can introduce the industrial food to the fish larvae (19 DAH). The last important day for the ontogeny of the digestive system of greater amberjack larvae was day 22 (11 mm TL) when the pyloric caeca appeared in the Copepods group. In the Control group, pyloric caeca appeared later, 28 days after hatching. Those days were also important for the ontogeny of the skeleton of the greater amberjack with the beginning (12 DAH) and completion (22 DAH) of the flexion (Figure 3).

**Figure 4:**
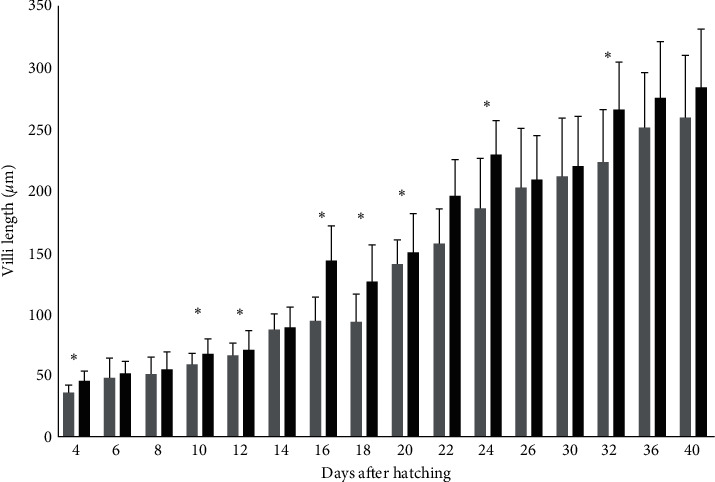
Length of the villi (*μ*m ± S.D., *n* = 6 ind./tank) in greater amberjack in both Control (grey bars) and Copepods (black bars) group. The days with statistically significant differences are denoted with an asterisk (*p*  < 0.05).

Besides the timing of appearance of the pyloric caeca, the two groups had differences in the length and surface of the villi and the abundance of goblet cells in the intestine as well as the percentage of area covered with lipid vacuoles in the liver. A common pattern was observed in all those indices. More specifically, the fish larvae from the Copepods group had higher villi length than the fish larvae from the Control group on days 4 (3.7 mm TL), 10 (4.5 ± 0.1 mm TL), 12 (5 mm TL), 16 (6.1 mm TL), 18 (7.4 ± 0.3 mm TL), 20 (8.9 ± 0.7 mm TL), 24 (12. 5± 0.5 mm TL), and 32 (18.2 ± 0.8 mm TL; *p*  < 0.05; Figure 4).

**Figure 5:**
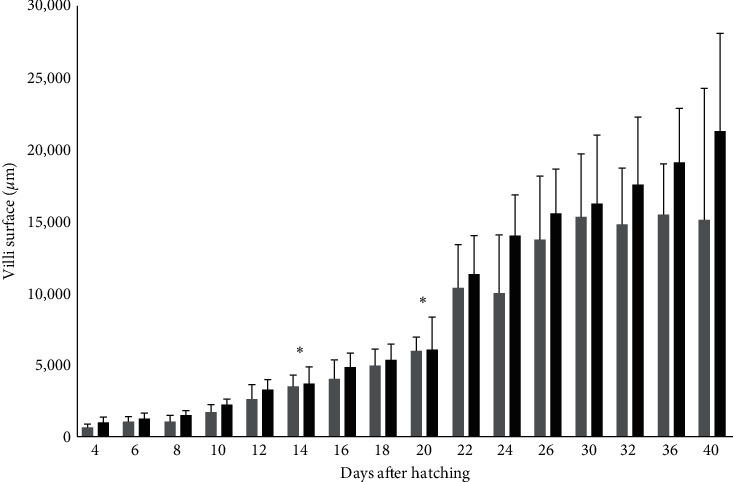
Surface of the villi (*μ*m ± S.D., *n* = 6 ind./tank) in greater amberjack in both Control (grey bars) and Copepods (black bars) group. The days with statistically significant differences are denoted with an asterisk. (*p*  < 0.05).

The surface of the villi had also similar results since it was higher in the fish larvae from the Copepods group on days 14 (5.6 ± 0.1 mm TL) and 20 (8.9 ± 0.7 mm TL; *p*  < 0.05; Figure 5).

**Figure 6:**
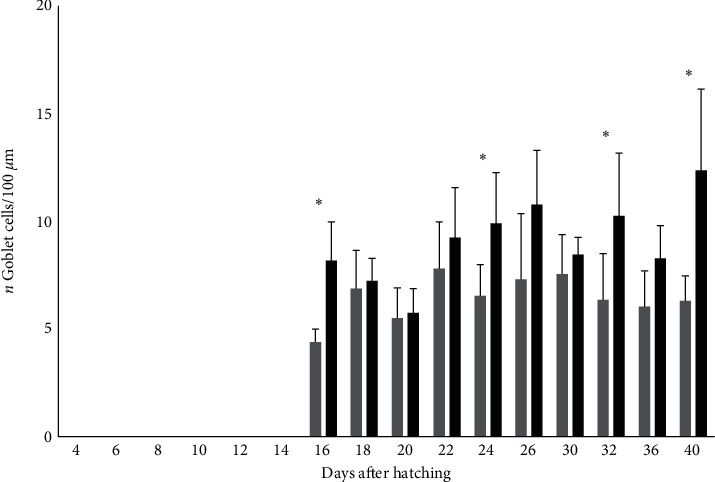
Number of goblet cells/100 *μ*m (ind ± S.D., *n* = 6 ind./tank) in greater amberjack in both Control (grey bars) and Copepods (black bars) group. The days with statistically significant differences are denoted with an asterisk. (*p*  < 0.05).

In the anterior intestine, we also measured the abundance of goblet cells/100 *μ*m. Goblet cells were observed for the first time at 15 DAH in both groups. The abundance of goblet cells was higher in the villi of the fish larvae from the Copepods group than in the larvae from the Control group on days 16 (6.1 mm TL), 24 (12.5 ± 0.5 mm TL), 32 (18.2 ± 0.8 mm TL), and 40 (Control group: 18 ± 1.3 mm TL, Copepods group: 22.6 ± 1 mm TL; *p*  < 0.05; Figure 6).

**Figure 7:**
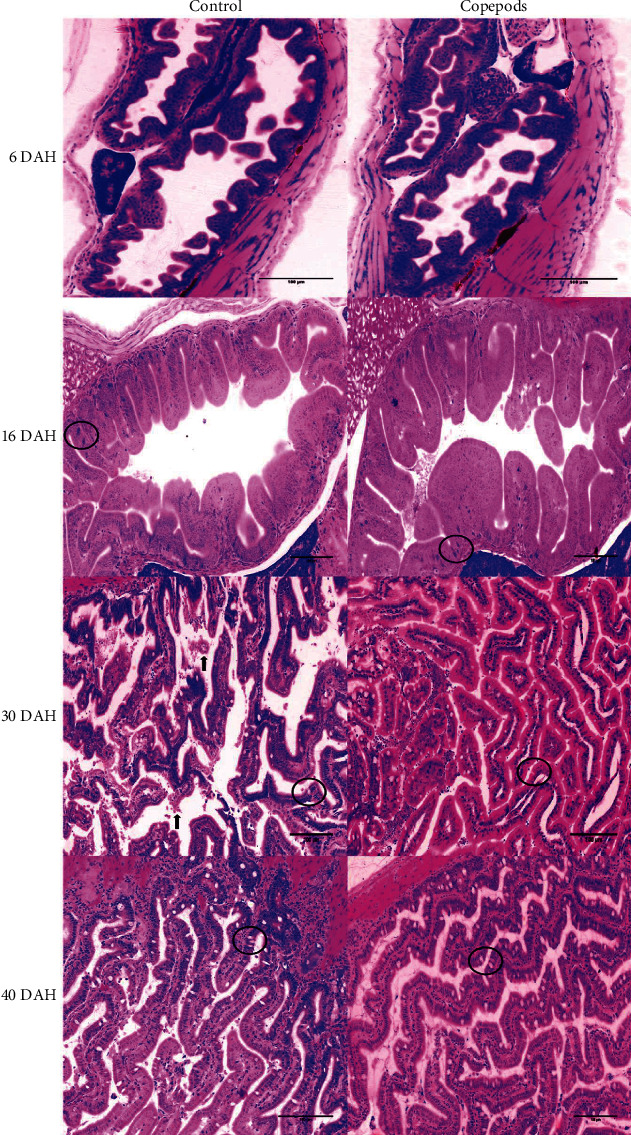
The ontogeny of the villi in the anterior intestine in greater amberjack in both Control and Copepods groups in 6, 16, 30, and 40 DAH. Black circles show the goblet cells and black arrows show the desquamation in the villi of the fish larvae of the Control group 30 DAH. Scale 100 *μ*m.

In addition, desquamation of the villi in the anterior intestine was observed in fish larvae from the Control group 30 DAH (17.3 ± 0.1 mm TL; Figure 7).

**Figure 8:**
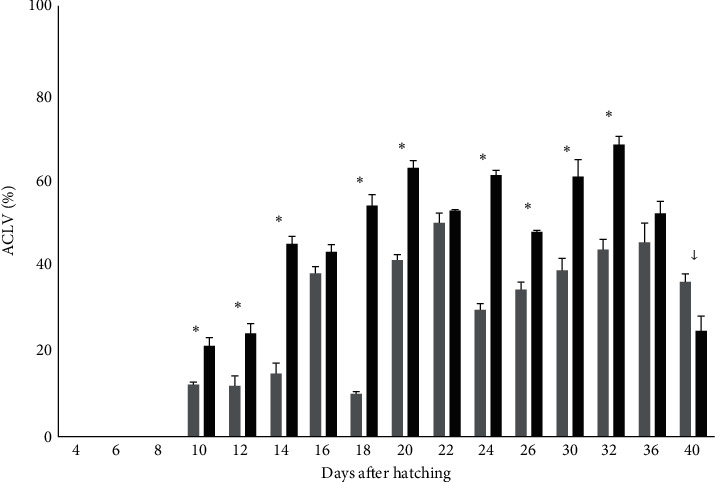
Area covered with lipid vacuoles (ACLV) in the liver (%, *n* = 6 ind./tank) in greater amberjack in both Control (grey bars) and Copepods (black bars) group. The arrow (↓) denotes the day when fish from the Control group were larger than fish from the Copepods group, asterisks ( ^*∗*^) show the days with statistically significant differences (*p*  < 0.05).

Regarding the liver, the area covered with lipid vacuoles was measured from 10 DAH. The percentage of ACLV was higher in the fish larvae from the Copepods group than the larvae from the Control group on days 10 (4.5 ± 0.1 mm TL), 12 (5 mm TL), 14 (5.6 ± 0.1 mm TL), 18 (7.4 ± 0.3 mm TL), 20 (8.9 ± 0.7 mm TL), 24 (12.5 ± 0.5 mm TL), 26 (18.8 mm TL), 30 (17.3 ± 0.1 mm TL), and 32 (18.2 ± 0.8 mm TL; *p*  < 0.05). The exception was the sampling on day 40 where the ACLV in the liver was higher in the Control group but the lipid vacuolated hepatocytes were organized in a focal area of cellular change with intense degeneration (Figure 8).

**Figure 9:**
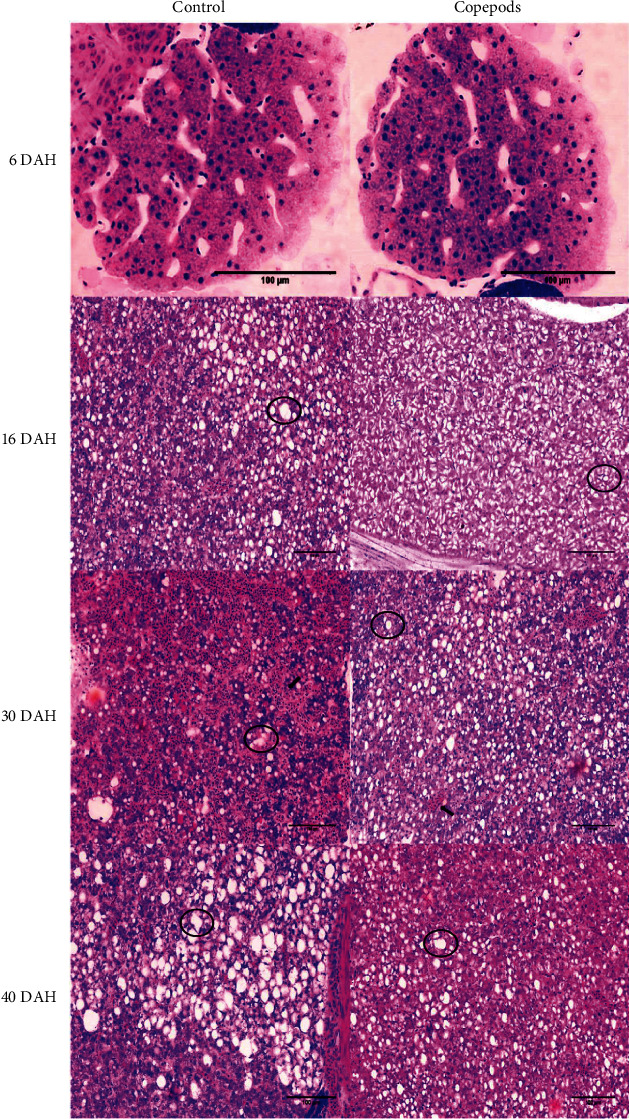
The ontogeny of the liver in greater amberjack in both Control and Copepods groups in 6, 16, 30, and 40 DAH. Black circles show the lipid vacuoles in the hepatocytes and black arrows show hyperemia 30 DAH.

It was also observed severe hyperemia in the liver of the fish larvae from the Control group at 30 DAH (17.3 ± 0.1 mm TL; Figure 9).

Normalization of the histological indices of the digestive system showed that villi length, abundance of goblet cells, and fat accumulation in the liver in fish larvae were affected by the use of copepods (*p*  < 0.05). On the other hand, the surface of the villi was more influenced by growth.

## 4. Discussion

### 4.1. Ontogeny of the Digestive System

Similar to other marine fishes, the organogenesis of the digestive system of the greater amberjack progressed in three phases, based on histological characteristics. The first phase began from the moment of hatching until 3 DAH, with the opening of the mouth. The duration of this phase in greater amberjack is similar to *Solea solea* [37], *S. lalandi* [6, 7], *S. rivoliana* [8], and *S. dumerili* [5] where the diet is based exclusively on the absorption of the nutrients of the yolk sac and the lipid droplets for the first 2 DAH, but it was shorter than *Dentex dentex* [38], and *Dicentrarchus labrax* [39], where nutrition was endogenous for four to 5 DAH. A rapid differentiation (3 DAH) is observed in the intestine due to the presence of the ileorectal valve that divides it into the middle and posterior parts. The appearance of the ileorectal valve occurs on 3 DAH in many other species, such as *Gadus morhua* [40], *Solea senegalensis* [41], *Diplodus sargus* [42], *Pagellus erythrinus* [43], *Pagrus pagrus* [44], *S. lalandi* [6, 7] and *S. rivoliana* [8]. On the contrary there are many other species where the appearance of ileorectal valve occurs later than in greater amberjack, *Sparus aurata* (4–6 DAH) [45, 46], *D. labrax* (5 DAH) [47], *D. dentex* (5 DAH) [38], and *S. dumerili* (4 DAH) [5]. By the end of the first phase, the initial hepatocytes and pancreatic cells had formed. The liver and pancreas are of endodermal origin [48] and appear very early in development. In greater amberjack, the liver begins to organize and function 3 DAH, as has been reported for other species, the *S. solea* [37], *Paralichthys dentatus* [49], *S. lalandi* [6, 7], and *S. rivoliana* [8].

The second phase is a transitional period where fish larvae develop mechanisms to adapt to exogenous feeding [50]. During this period (4–10 DAH), in the digestive system of greater amberjack larvae, the cardiac and pyloric sphincter (4 DHA) in the stomach appeared. The pyloric sphincter in *S. dumerili* in the study of Pérez et al. [5] appeared on 7 DAH in both intensive and mesocosm systems. The rapid increase in the length of the intestine, with its first coil was already created 4 DAH and it helps to increase the retention of food in the intestine, as well as in the improvement of digestion processes and absorption. The development of the villi indicates an increase in the absorptive surface of the intestine. In addition, fat deposition in the posterior intestine has occurred. In the second phase, the differentiation of hepatocytes and pancreatic cells began. The presence of glycogen at the end of this phase coincided with the differentiation of liver cells, following the same pattern as other species, such as *D. labrax* [51], *D. dentex* [38], *S. lalandi* [6, 7], and *S. rivoliana* [8]. By the end of the second phase, greater amberjack larvae had taste glands in the buccopharynx (6 DAH) and folds and goblet cells in the esophagus (6 and 7 DAH). The timing of the appearance of goblet cells in the esophagus is similar in other species such as *S. lalandi* (6–7 DAH) [6, 7], *S. rivoliana* (6–7 DAH) [8] *Pagrus pagrus* (6 DAH) [44], *D. sargus* (6 DAH) [42], *S. aurata* (7–8 DAH) [45], *D. labrax* (5–9 DAH) [51],, and *D. dentex* (7 DAH) [38]. In some other species like *S. solea* (2 DAH), and *S. senegalensis* (3 DAH) [37, 41] the goblet cells in the esophagus appeared earlier than in *G. morhua* (11 DAH) [38], and *S. dumerili* (13 DAH) [5].

The third phase of the organogenesis of the digestive system of amberjack signalls the appearance of pharyngeal teeth (12 DAH) and gastric glands in the stomach (14 DAH). The appearance of gastric glands signaled the formation of a functional stomach [52]. The timing of the appearance of gastric glands is the same in *D. sargus* [42], *S. lalandi* [6, 7] and *S. rivoliana* [8]. In the majority of the species in the aquaculture industry, the gastric glands appear later, *G. morhua* (52 DAH) [40], *S. senegalensis* (25–30 DAH) [41], *S. aurata* (45 DAH) [45, 46], *D. labrax* (55 DAH) [47], *D. dentex* (22 DAH) [38], *P. erythrinus* (28 DAH) [43], *Pagrus pagrus* (26–30 DAH) [44], and *S. dumerili* (17–20 DAH) [5]. By the end of this phase, goblet cells in the intestine had appeared (15 DAH). Species such as *D. labrax* [47] and *D. sargus* [42] follow this exact pattern regarding the timing of goblet cells appearance. There are many species in which the appearance of the goblet cells occurs later, such as *G. morhua* (62–70 DAH) [40], *D. dentex* (18 DAH) [38], and *P. erythrinus* (33 DAH) [43]. Meanwhile, in *S. solea* [37], *S. senegalensis* [41], it was found that the appearance of goblet cells in the intestine occurs very early (3 DAH). The formation of pyloric caeca (28 DAH) indicated the end of the third period and the completion of the organogenesis of the digestive system and is a major change in the ability of the larvae to digest food [49]. The differences in the appearance of some structures in the digestive system in greater amberjack between the present work and the study of Pérez et al. [5] can also result from the different conditions that prevail in each rearing and can affect the ontogeny of the digestive system.

### 4.2. Copepods Group

The order of appearance of the organs of the digestive system of greater amberjack was the same in both the Control and the Copepods group. Except for the appearance of pyloric caeca, the timing of those developmental stages was also the same. In the Copepods group, pyloric caeca appeared on 22 DAH, while in the fish larvae from the Control group on 28 DAH. The pyloric caeca increase the area for digestion inside the limited space of the abdominal cavity [53], and thus they are one of the most important organs regarding the digestion of food. This period (22–28 DAH) was very important for the greater amberjack since it was the weaning period (the formulated diet was given 25 days after hatching and the fish from the Copepods group already developed an extra organ (pyloric caeca) for better digestion and absorption of the food. The weaning period may result in low survival rates and growth, due to decreased feeding rates of the larvae since the formulated diet results in lower feed ingestion compared with live food [13]. Furthermore, the digestive system of the fish must be fully organized before introducing a formulated diet for the amount consumed by the fish to be absorbed and used for their growth. The appearance of the pyloric caeca signals the formation of a fully functional digestive system. Fish with functional stomach and intestine can break down proteins and absorb amino acids by the action of pepsin [54]. The development of the villi in the intestine could also affect the digestion ability. It was found that the length and the surface of the villi of the fish from the Copepods group were higher than the fish from the Control group. Bigger villi offer extra space for digestion and thus better absorption of nutrients.

Another important difference in the ontogeny of the greater amberjack larvae of the two groups was that the abundance of goblet cells in the intestine was higher in the Copepods group. The mucus secreted by goblet cells in the intestine in addition to lubricating the digestive tract and facilitating the passage of ingested particles [55–57], participates also in the process of osmoregulation [58] since the quality of intestinal mucous substances is directly linked to environmental conditions which affect the function of the digestive tract [59]. The presence of mucus is likely to regulate the transportation of proteins, or to ensure their further processing, in a biochemical way, thanks to the various ions they contain [46, 54]. The higher quantity of mucus in the larvae from the Copepods group protects their intestinal segment during periods of poor nutrition from existing enzymes that might cause autolysis in the intestinal tissues and contribute positively to digestion processes [57]. The higher length and surface of the villi, higher abundance of goblet cells and the earlier presence of the pyloric caeca in the fish larvae from the Copepods group resulted in additional area for digestion and better absorption of nutrients. Better digestive capacity could explain the difference in the total length where in the last days the fish from the Copepods group were bigger than the larvae from the Control group.

Another important difference in the ontogeny of the digestive system between the two rearing protocols was that the fat accumulation in the liver was higher in the Copepods group. From the beginning of the heterotrophic stage, fat deposition in the liver undergoes significant variations, which are affected by the quantitative and qualitative composition of available food, the developmental stage of larvae, and the presence of functional organs that produce the necessary enzymes for digestion and absorption of food. The liver is the organ that responds directly to any changes related to nutrition and the variation in liver fat indicates the response to the different types of food it consumes [60]. Therefore, the qualitative composition of copepods is higher than that of enriched rotifers. The lipid vacuoles in the liver are very important, especially during the weaning period and their morphology in the cytoplasm is related to the nutritional status of the larvae [6, 61]. The weaning period can be very stressful for the fish since until they adapt to the formulated diet, they go through a period of poor nutrition that can have negative effects on their growth and survival. Lipid vacuoles in the liver are energy reserves for periods where the food is limited, or the diet is not optimal. In the present study, it was found that throughout most days of the weaning period, the fish from the Copepods group had statistically significantly higher areas covered with lipid vacuoles in the liver than the fish from the Control group. The reduction of the fat accumulation in the liver of the fish from the Copepods group during the last two samplings could be explained by its use for body growth and is reflected in the significantly higher total length of those fish in those last days of the trial.

The test to determine whether the more developed digestive system of the copepod-fed fish was influenced by the effect of growth or was a direct result of the use of copepods showed that in the cases of villi length, the abundance of goblet cells and ACLV in the liver, copepods had an immediate positive effect. On the contrary, the increase in the surface of the villi was mostly affected by growth. As in all tanks of both groups, the rearing conditions were stable, and the only variable factor was the use or not of copepods. The increase in total length in the fish from the Copepods group can be attributed to the two different feeding protocols.

Both the length and surface of the villi showed greater differences (statistically significant differences) between the feeding protocols up to 20 DAH where both had higher values in the Copepods group throughout the whole rearing period. The fact that the period in which these differences were more pronounced (4–20 DAH) largely coincides with the period of use of copepods (3–17 DAH) proves that the use of copepods enhances the growth of villi. In addition, it was proven that the copepod-fed fish had higher total body length and the normalization of the data showed that the higher body length also affected the surface area of the villi. However, given that we are referring to the same structure (villi of the intestine), the question arises as to why the length was directly affected by the use of the copepods while the surface was indirectly affected. The answer to this question could lie in the growth rate of the villi. The total length of the fish as well as the surface of the villi show an exponential increase as both these values increase over time, and both had higher values at each subsequent sampling. On the contrary, the length of the villi did not show an exponential growth rate but fluctuated, since we can observe variations in these values (16, 26, and 30 DAH). This fact confirms that the body length increases in parallel with the surface of the villi which it affects, while the length of the villi seems not to be affected by the body length but exclusively by the diet.

The use of copepods during the first days of rearing improved the ontogeny of the digestive system mainly due to the higher nutritional profile of copepods compared with rotifers. Both free amino acids and fatty acids, in addition to their beneficial properties for the digestive system, enhance the foraging behavior of the fish. More specifically, free amino acids enhance the sense of smell while fatty acids improve the development of the nervous system and thus the retina [9, 18]. It is, therefore, possible that the fish larvae from the Copepods group developed better feeding behavior related to foraging through both olfaction and vision and thus were able to hunt and consume higher amounts of food. This could explain not only the better ontogeny of the digestive system but also the difference in total length in the last days of rearing. However, copepods are not only beneficial for the development of the digestive system. From 10 to 17 days after hatching copepod-fed larvae had significantly lower mortality rates than the fish from the Control group. Fish from the same experiment were analyzed also to study the effect on the ontogeny of the skeleton. More specifically, it was found that both the appearance of the structures (cartilages and bones), the completion of their appearance (achievement of the final number of elements in a structure) and the completion of calcification were improved in the fish fed with copepods. In addition, the number and severity of deformities were higher in the fish from the Control group, which is possible to affect also the mortality rate [62].

## 5. Conclusions

The results of the present study showed that the addition of copepods nauplii in the feeding protocol of the greater amberjack larvae had a positive effect on the ontogeny of the digestive system. More specifically, it was found that fish larvae from the Copepods group had a larger area for digesting food and absorbing nutrients due to the early appearance of pyloric caeca and larger villi in terms of both length and surface. The increased number of goblet cells and, by extension, the produced mucus in the copepod-fed larvae, also contributed to easier digestion and more efficient absorption of nutrients from the food. The mucus also amplified their immune system and combined with the higher percentages of stored fat in the liver, protected the fish from periods of poor nutrition, such as the weaning period. The reduction of the fat accumulation in the liver of the fish from the Copepods group during the last two samplings could be explained by its use for body growth and is reflected in the significantly higher total length of those fish.

## Figures and Tables

**Table 1:** Vitamins, minerals, and fatty acids content of the different feeds used in the first days of larval rearing of greater amberjack.

	Rotifers	*Artemia* nauplii	Enriched *Artemia* metanauplii	Copepods	Formulated diet
Vitamins (mg kg^−1^ DW)					
Vitamin C	117	400	798	500	1.7
Vitamin E	85	64	70	110	0.4
Minerals (mg kg^−1^ DW)					
Iron	84	Na	63	371	—
Selenium	0.08	Na	2.2	5	1.5
Zinc	62	Na	120–310	340	—
Copper	2.7	Na	7	12	—
Magnesium	3,900	Na	4,000	8,000	—
Calcium	1,900	Na	1,900–2,000	2,400	1,500
Fatty acids (% of total FA)					
20 : 5n3 (EPA)	6.16 ± 0.04	Na	12.55 ± 1.03	17.7 ± 10.79	6.97
22 : 6n3 (DHA)	17.1 ± 0.13	Na	18.08 ± 0.31	28.71 ± 10.58	10.8
Sum n3 fatty acids	26.73 ± 0.18	Na	35.73 ± 1.14	59.13 ± 7.51	21.17
Sum n6 fatty acids	7.98 ± 0.03	Na	10.21 ± 1.82	11.35 ±5.57	28.19
Total lipids (% of dry weight)	16.25 ± 1.72	Na	21.28 ± 1.82	9.14 ± 0.74	10.4

Na: not available. Data from [31, 33, 34].

**Table 2:** Feeding and sampling schedule during the greater amberjack start-feeding experiment.

	Copepods group		Control group			Formulated diet		Samplings	
DAH	Rotifer (feedings/day × ind/mL)	Copepod nauplii (feedings/day × ind/mL)	Rotifer (feedings/day × ind/mL)	*Artemia* nauplii (feedings/day × ind/mL)	*Artemia* metanauplii (feedings/day × ind/mL)	Size 100/200 (g)	Size 200/300 (g)	Histology (*n*/tank)	Total length (*n*/tank)
1	—	—	—	—	—	—	—	—	15 ind.
2	—	—	—	—	—	—	—	—	15 ind.
3	6×20	6 × 35	6 × 50	—	—	—	—	—	15 ind.
4	6×20	6 × 35	6 × 50	—	—	—	—	6 ind.	15 ind.
5	6 × 20	6 × 35	6 × 50	—	—	—	—	—	15 ind.
6	6 × 20	6 × 35	6 × 50	—	—	—	—	6 ind.	15 ind.
7	6 × 20	6 × 35	6 × 50	—	—	—	—	—	15 ind.
8	6 × 20	6 × 35	6 × 50	—	—	—	—	6 ind.	15 ind.
9	6 × 20	6 × 35	6 × 50	—	—	—	—	—	15 ind.
10	6 × 20	6 × 35	6 × 50	6 × 1.5	—	—	—	6 ind.	15 ind.
11	6 × 20	6 × 35	6 × 50	6 × 1.5	—	—	—	—	15 ind.
12	6 × 20	6 × 35	6 × 50	6 × 1.5	—	—	—	6 ind.	15 ind.
13	6 × 20	6 × 35	6 × 50	6 × 1.5	—	—	—	—	15 ind.
14	6 × 20	6 × 35	6 × 50	6 × 1.5	—	—	—	6 ind.	15 ind.
15	6 × 20	6 × 35	6 × 50	6 × 1.5	—	—	—	—	15 ind.
16	6 × 20	6 × 35	6 × 50	6 × 1.5	—	—	—	6 ind.	15 ind.
17	6 × 20	6 × 35	6 × 50	6 × 1.5	—	—	—	—	15 ind.
18	6 × 35	—	6 × 35	6 × 2	—	—	—	6 ind.	15 ind.
19	6 × 35	—	6 × 35	6 × 2	—	—	—	—	15 ind.
20	6 × 20	—	6 × 20	6×2	6 × 2	—	—	6 ind.	15 ind.
21	6 × 20	—	6 × 20	6 × 2	6 × 2	—	—	—	15 ind.
22	6 × 20	—	6 × 20	6 × 2	6× 2	—	—	6 ind.	15 ind.
23	6 × 20	—	6 × 20	—	6 × 2	—	—	—	15 ind.
24	6 × 20	—	6 ×20	—	6 × 2	—	—	6 ind.	15 ind.
25	6 × 20	—	6× 20	—	6 × 2	m.f. × 45	—	—	15 ind.
26	6 × 20	—	6 × 20	—	6 × 2	m.f. × 45	—	6 ind.	15 ind.
27	6 × 20	—	6 × 20	—	6 × 2	m.f. × 45	—	—	15 ind.
28	—	—	—	—	6 × 2	m.f. × 45	—	—	15 ind.
29	—	—	—	—	6 × 2	m.f. × 20	m.f. × 40	—	15 ind.
30	—	—	—	—	6 × 2	m.f. × 20	m.f. × 40	6 ind.	15 ind.
31	—	—	—	—	—	m.f. × 15	m.f. × 30	—	15 ind.
32	—	—	—	—	—	m.f. × 15	m.f. × 30	6 ind.	15 ind.
33	—	—	—	—	—	m.f. × 15	m.f. × 30	—	15 ind.
34	—	—	—	—	—	m.f. × 15	m.f. × 30	—	15 ind.
35	—	—	—	—	—	m.f. × 15	m.f.×30	—	15 ind.
36	—	—	—	—	—	m.f. × 15	m.f.×30	6 ind.	15 ind.
37	—	—	—	—	—	m.f. × 15	m.f. × 30	—	15 ind.
38	—	—	—	—	—	m.f. × 15	m.f. × 30	—	15 ind.
39	—	—	—	—	—	m.f. × 15	m.f. × 30	—	15 ind.
40	—	—	—	—	—	m.f. × 15	m.f. × 30	6 ind.	15 ind.

m.f.: manually frequently.

## Data Availability

All data are presented in this manuscript.
